# Gene expression profiling reveals the effects of light on adventitious root formation in lotus seedlings (*Nelumbo nucifera* Gaertn.)

**DOI:** 10.1186/s12864-020-07098-5

**Published:** 2020-10-12

**Authors:** Cheng Libao, Han Yuyan, Zhao Minrong, Xu Xiaoyong, Shen Zhiguang, Wang Chunfei, Li Shuyan, Hu Zhubing

**Affiliations:** 1 School of Horticulture and Plant Protection, Yangzhou University, Yangzhou, Jiangsu P. R. China; 2 Key Laboratory of Plant Stress Biology, School of Life Sciences, Henan University, Kaifeng, 475004 China; 3 Henghui Food Co., Ltd of Yancheng, Kaifeng, 224700 China; 4 College of Guangling, Yangzhou University, Yangzhou, Jiangsu P. R. China

**Keywords:** Lotus, ARs, Light, Gene, IAA, Sucrose

## Abstract

**Background:**

Lotus is an aquatic horticultural crop that is widely cultivated in most regions of China and is used as an important off-season vegetable. The principal root of lotus is degenerated, and adventitious roots (ARs) are irreplaceable for plant growth. We found that no ARs formed under darkness and that exposure to high-intensity light significantly promoted the development of root primordia. Four differential expression libraries based on three light intensities were constructed to monitor metabolic changes, especially in indole-3-acetic acid (IAA) and sugar metabolism.

**Results:**

AR formation was significantly affected by light, and high light intensity accelerated AR development. Metabolic changes during AR formation under different light intensities were evaluated using gene expression profiling by high-throughput tag-sequencing. More than 2.2 × 10^4^ genes were obtained in each library; the expression level of most genes was between 0.01 and 100 (FPKF value). Libraries constructed from plants grown under darkness (D/CK), under 5000 lx (E/CK), and under 20,000 lx (F/CK) contained 1739, 1683, and 1462 upregulated genes and 1533, 995, and 834 downregulated genes, respectively, when compared to those in the initial state (CK). Additionally, we found that 1454 and 478 genes had altered expression in a comparison of libraries D/CK and F/CK. Gene transcription between libraries D/F ranged from a 5-fold decrease to a 5-fold increase. Twenty differentially expressed genes (DEGs) were involved in the signal transduction pathway, 28 DEGs were related to the IAA response, and 35 DEGs were involved in sugar metabolism. We observed that the IAA content was enhanced after seed germination, even in darkness; this was responsible for AR formation. We also observed that sucrose could eliminate the negative effect of 150 μMol IAA during AR development.

**Conclusions:**

AR formation was regulated by IAA, even in the dark, where induction and developmental processes could also be completed. In addition, 36 genes displayed altered expression in carbohydrate metabolism and ucrose metabolism was involved in AR development (expressed stage) according to gene expression and content change characteristics.

## Background

Lotus is widely cultivated in the southern region of the Yellow River Basin; a lotus cultivation area of approximately 200,000 ha is mainly distributed in Hubei, Jiangsu, Anhui, Guangdong, and Shandong provinces. The lotus is commonly used for three main purposes: lotus flowers can be used for ornamental displays, lotus rhizomes can be used as vegetables, and lotus seed can be used as a food source. Lotus rhizome can be continuously supplied to the local market as a vegetable owing to its simple storage in soil from October to April of the next year. Traditional cuisine such as steamed lotus, boiled lotus, and lotus soup are very popular among consumers. Several processed parts of lotus plants, including lotus starch, lotus drink, and salted lotus, are exported to a number of countries in Asia, America, and Europe [1]. In addition, a large number of secondary metabolites make the lotus a constituent of traditional Chinese medicine. Recently, with greater industrialization, lotus cultivation has increased to cover the largest area among all the aquatic vegetables. Therefore, studies related to the theory and practice of lotus use have been attracting increasing attention [2, 3].

Light, including photoperiod, light quality, and light intensity, is a basic condition that is involved in several aspects of plant development and growth, such as root formation, photosynthesis, flowering, fruit development, and plant morphogenesis [4–6]. Many metabolic processes that depend on light signals during plant growth are induced by hormonal signaling [7, 8], suggesting that hormone action occurs downstream of the light signal transduction pathway [9–11]. Light is known to regulate the entire process of root formation [12–14]. A number of factors involved in the light signaling pathway, such as reactive oxygen species, abscisic acid, and sugar, have been to found to affect root development [15–17]. Indole-3-acetic acid (IAA) is synthesized in vigorous organs under light regulation [18, 19]. Depending on the exposure to light, IAA plays a critical role in the developmental process of adventitious roots (ARs), including induction, development, and expression of roots [20, 21]. Improvement of endogenous IAA content by exogenous application of IAA significantly promotes cell division of root primordium, which directly leads to a positive effect on AR development [22]. Further, studies show that changing auxin metabolism or auxin sensitivity in plants is helpful for the formation of ARs [23, 24]. It has been reported that cytokinin, which regulates cell division, is also involved in AR formation due to its effect on auxin metabolism [20]. Therefore, IAA is considered a direct regulator of the complex network in regulating AR formation.

Analysis of gene expression or regulation in the whole genome is the most effective approach to understand AR formation. Studies over recent decades involved in auxin metabolism or responses related to ARs have shown that many genes participate in IAA synthesis, transport, or response which help accelerate developmental processes of ARs [25]. Until now, two kinds of IAA transport (influx carriers and efflux carriers) have been reported. The AUX1/LAX family, which are influx carriers, has a major influence on root development by triggering IAA distribution in plants [26]. The AUX1/ LAX gene family contains several members, and different expression profiles are found in various tissues [27]. Ahkami et al. (2013) [28] reported that auxin also affects the IAA content in plants by regulating *GH3* expression in *Petunia hybrida*. The above data indicate that various functions exist for members of the AUX1/LAX family, although these genes are involved in AR development. PIN, as an efflux carrier*,* is expressed in the root primordia and is required for root formation [29, 30]. An auxin-induced gene, ARL1, is found to participate in cell division relevant to AR formation [25]. In addition, several auxin responsive factors, such as ARF6, ARF8, and ARF17, are also involved in AR development [31]. Therefore, the biological process of ARs formation is regulated by multiple genes.

Lotus needs considerable nutrition to support plant growth. However, the principal root cannot be developed in the plant owing to long periods of evolution; therefore, ARs become the major mediators for uptake of water and mineral substances for adequate swelling of rhizome, which is essential for production or breeding of lotus. Recently, we have found that ARs of seedling hypocotyl significantly affect plant growth. Early formation of ARs or more ARs number can promote swelling of rhizome. The ARs of lotus are primordially latent and need to be induced by IAA [32] for the developmental process to start. ARs have been found to frequently locate in two sites in lotus plants, namely the seedling hypocotyl and the internodes of storage organs [33]. In general, the number of ARs in the seedling hypocotyl is lower than that in the internodes of storage organs owing to the considerable amount of nutrition that is needed for plant growth. Primordial roots are differentiated from normal cells triggered by hormones or other environmental factors and developed at the pericycle [34, 35]. The biological process of AR formation includes three periods: induced stages, initial developmental stages, and emergence from the epidermis [36, 37]. In the induced stage, meristematic cells are developed from normal cells; the sink establishment phase is thus established. In the initial developmental stage, the primordium relevant to ARs is formed and developed [38], and finally, ARs protrude from the epidermis [39]. The above three biological processes are affected by light.

Recently, we found that exogenous application of ethylene, IAA, and mechanical damage significantly affected lotus AR formation derived from the change in endogenous IAA content under normal light conditions. In darkness, no emergence of ARs occurs, although the above substances were applied, suggesting that light is a necessary factor for lotus AR development. However, there was no direct evidence for the light-dependent IAA regulation on AR formation. Therefore, in this study we constructed four gene libraries to monitor gene expression from the induced stage to the expression stage of AR development. At the same time, changes in IAA content were also documented.

## Results

### Light promotes AR development

**Fig. 1:**
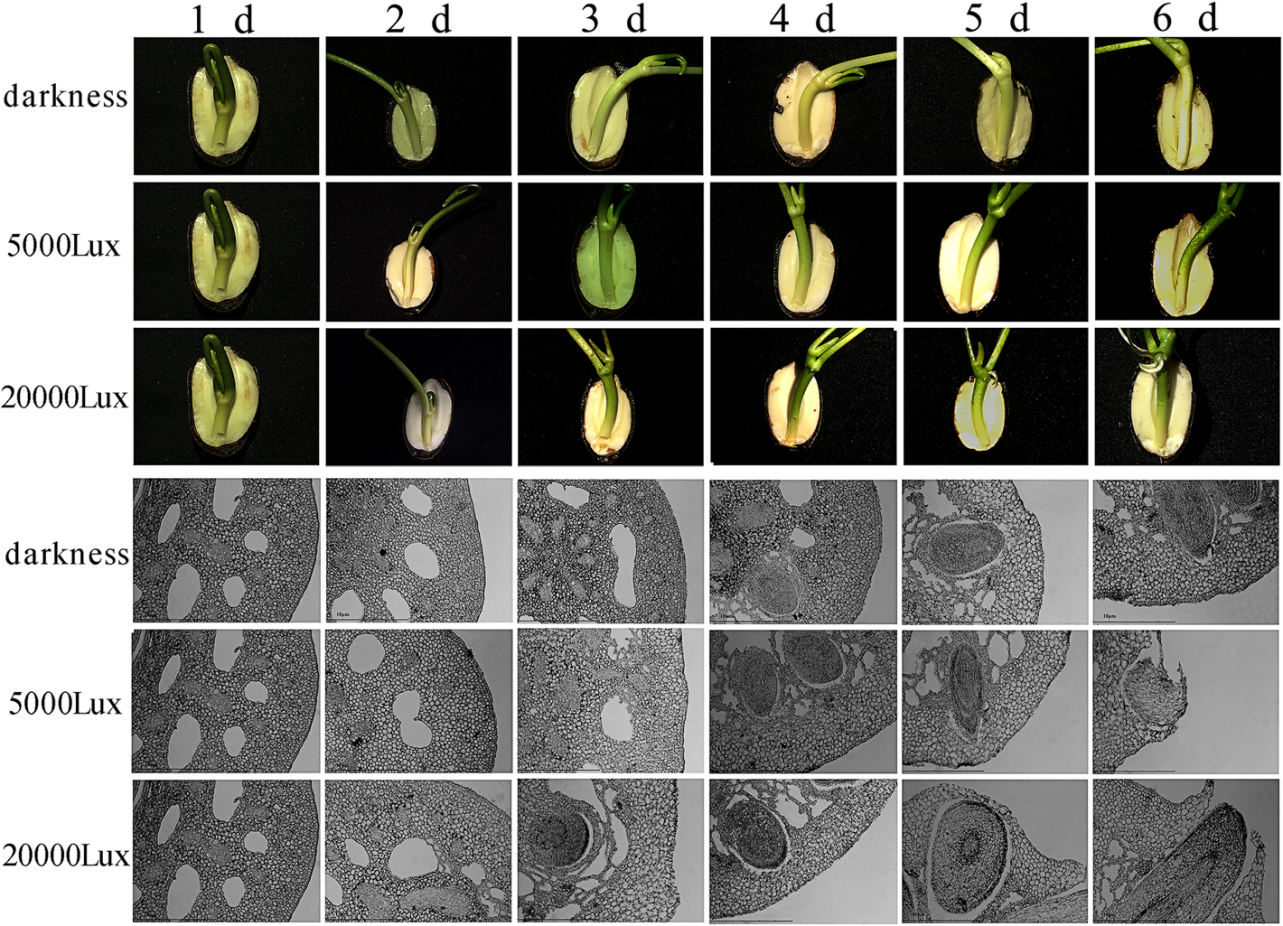
Changes in morphology and microstructure of ARs after treatment with various light intensities. **a** Changes in the morphology of ARs in lotus under darkness, and under 5000 and 20,000 lx light intensities over 5 days. **b** Changes in the microstructure of ARs in lotus under darkness, and under 5000 and 20,000 lx light intensities over 5 days

To investigate the effect of light quality on lotus AR formation, lotus was exposed to various light intensities, including darkness, and 5000 and 20,000 lx. No ARs were formed in the lotus under darkness (Table 1), whereas lotus could develop ARs when exposed to light. Thus, AR formation appears to be dependent on light intensity. After germination, ARs could be observed on the second day under 20,000 lx and on the fourth day under 5000 lx, indicating that light regulates AR development (Fig. 1a). Next, we observed the microstructure of the hypocotyl where the ARs emerged. When exposed to light, AR development could be clearly divided into three stages: induced process, developmental process, and expressed process (Fig. 1b). Under darkness, the AR primordium was present, but failed to break out of the epidermis (Fig. 1b).

**Table. 1:** Effect of various light intensities on the number and rates of AR

Treatments	1 d		2 d		3 d		4 d		5 d		6 d	
	AN	AR(%)	AN	AR(%)	AN	AR(%)	AN	AR (%)	AN	AR (%)	AN	AR (%)
Darkness	0c	0c	0c	0c	0b	0c	0d	0d	0c	0c	0c	0c
5000 lx	0.53b	18b	0.61c	43b	2.86c	67b	4.33c	77b	5.63b	88a	7.84b	94a
30,000 lx	1.53a	51a	4.32a	85a	4.57a	94a	8.83a	97a	10.72a	98a	12.34a	100a

### Light affects IAA content

**Fig. 2:**
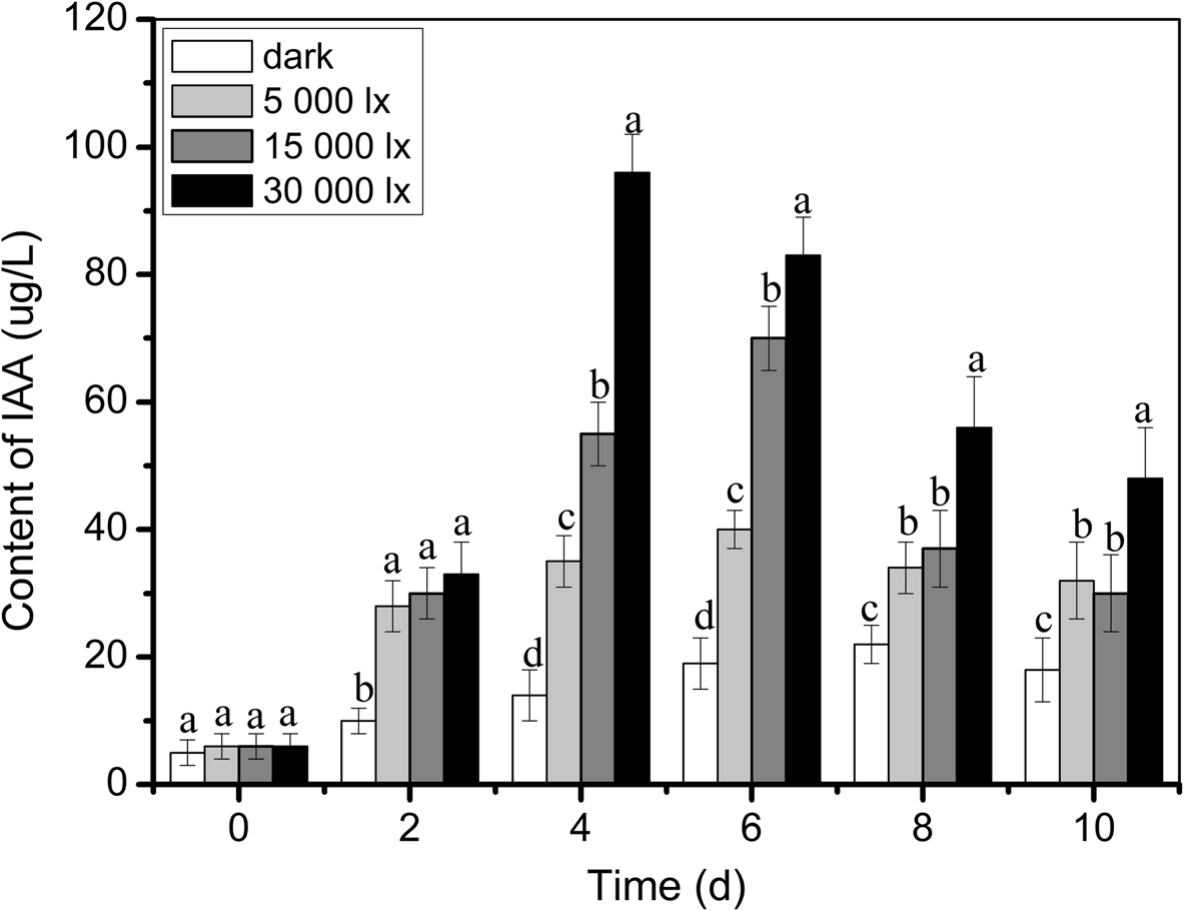
IAA and sucrose content during AR development. **a** IAA content at 0, 2, 4, 6, 8, and 10 d after treatment under darkness, and under 5000, 15,000, and 30,000 lx light intensities in lotus seedlings. **b** Sucrose content at 0, 2, 4, 6, 8, and 10 d after treatment under darkness, and under 5000, 15,000, and 30,000 lx light intensities in lotus

IAA has been characterized as an inducer of ARs. To investigate whether the regulation of light on ARs is dependent on IAA, we monitored the IAA contents of lotus seedlings under various light intensities (darkness, 5000, 1500, and 30,000 lx). IAA content gradually increased with exposure time to light and reached a maximum within 4 days and subsequently decreased; interestingly, a significant increase in IAA content was also observed in darkness. Among the different light intensities, the increased level of IAA in lotus was the highest under 30,000 lx. The above results showed that another factor, which was regulated by light, existed in coordinating the development of ARs with IAA, (Fig. 2).

### Effects of light on transcriptome profiling

**Fig. 3:**
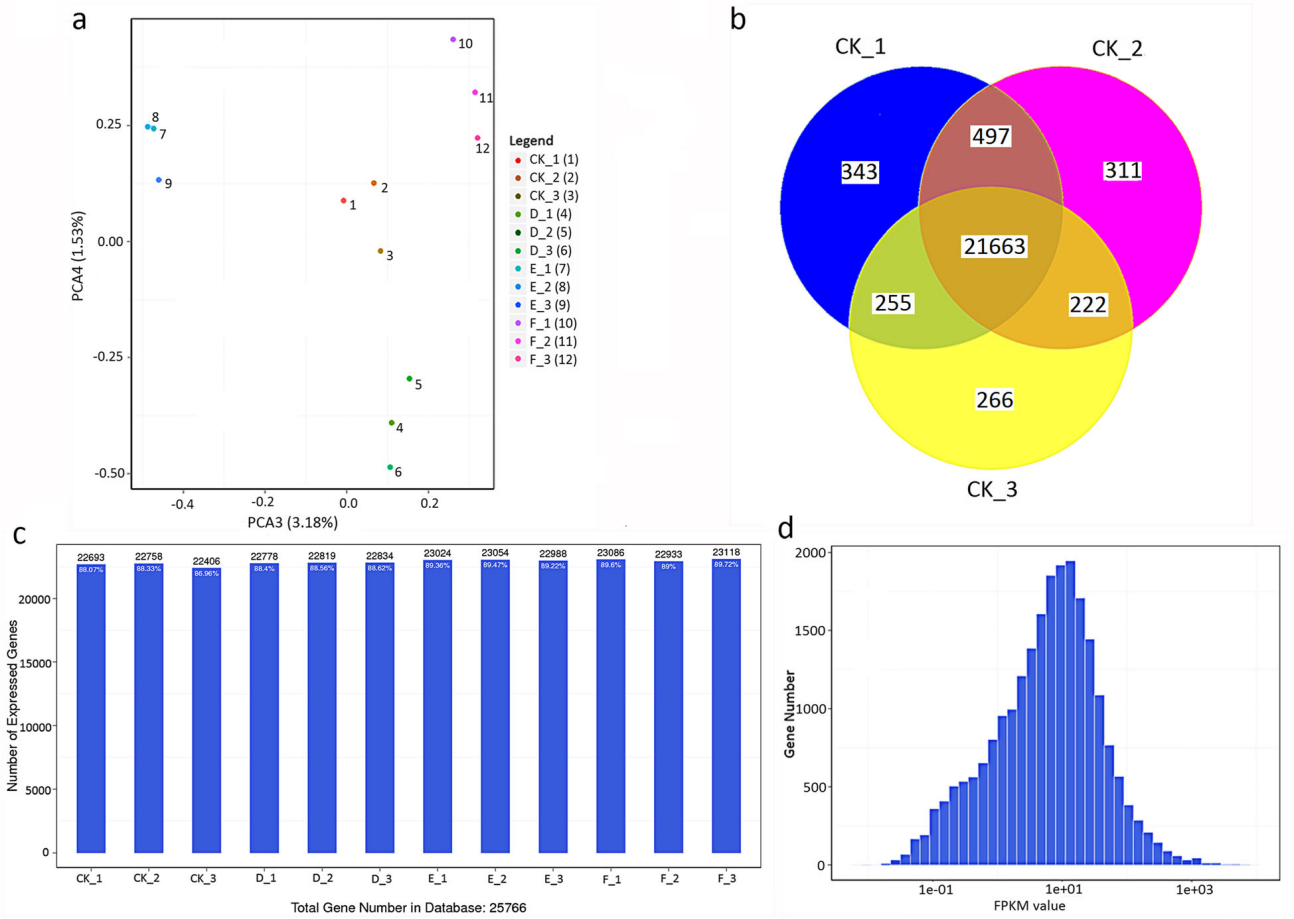
Essential data derived from the RNA-seq technique. **a** The result of principal component analysis between the components in libraries. **b** Venn Chart of co-expressed genes among the repeated samples. **c** Number of identified genes in all libraries. **d** Histogram distribution of genes on the expression level of each sample

**Fig. 4:**
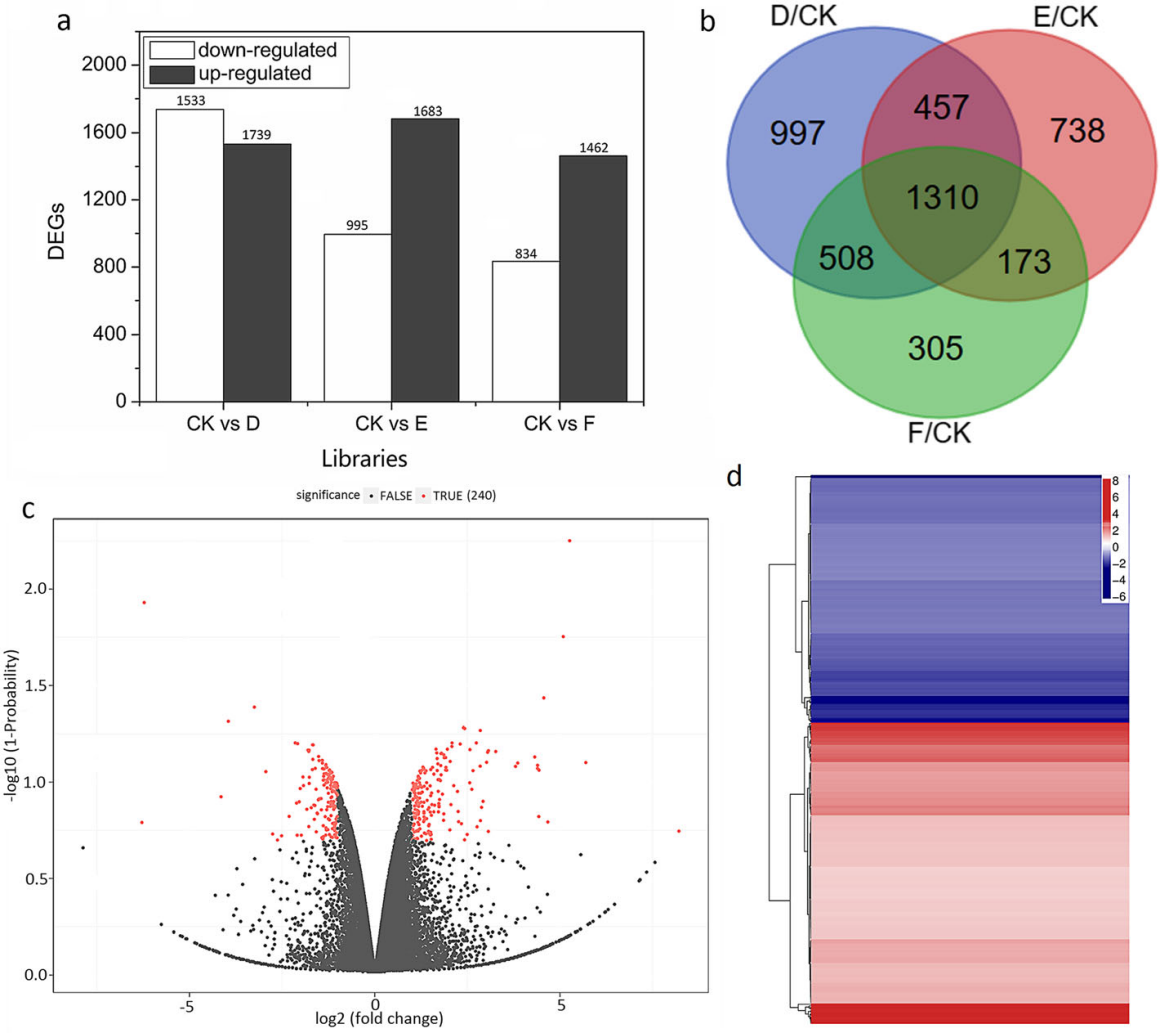
Identification of differentially expressed genes under different light intensities. Number of DEGs in the D/CK, E/CK, and F/CK libraries. **b** Selected expressed genes in the D/CK, E/CK, and F/CK libraries. **c** Identification of DEGs in F/D libraries. **d** Distribution of expression of these DEGs identified in the F/D libraries

To dissect the underlying mechanism by which light regulates AR development, we comparatively analyzed the transcriptome profile of lotus before and after exposure to various light intensities (darkness, and 5000 and 20,000 lx) by constructing four different libraries: CK0 (before treatment), D (3-d exposure under darkness), E (3-d exposure under 5000 lx), and F (3-d exposure under 20,000 lx). Analysis of quality control showed that the reads derived from RNA-seq libraries covered the whole lotus genome, as evidenced by the flat curve of the obtained reads (Additional file 1: Fig. S1). Approximately 1.2 × 10^9^ reads were obtained, of which more than 97% were clean reads. Approximately 83% reads were successfully mapped into the lotus genome and 73% of the reads were unique (Additional file 1: Table S1). PCA showed a high correlation among the three biological replicates (Fig. 3a). In total, 25,766 genes were obtained, and over 86% of genes were present in each library (Fig. 3b). The FPKM values ranged from 0.01 to 100 (Fig. 3c,d). Analysis of differentially expressed genes (DEGs) showed that when compared with the expression before treatment (in library CK0), 1739, 1683, and 1462 genes were upregulated and 1533, 995, and 834 genes were downregulated when lotus plants were exposed to 3 days under darkness (in library D), or under 5000 lx (library E) or 20,000 lx (library F), respectively, (Fig. 4a,b, Additional file 1: file S). Further DEGs analysis between libraries D and F, only 240 genes satisfied the threshold of a DEG (Fig. 4c,d).

### Light influences carbohydrate metabolism and hormone signal transduction

**Fig. 5:**
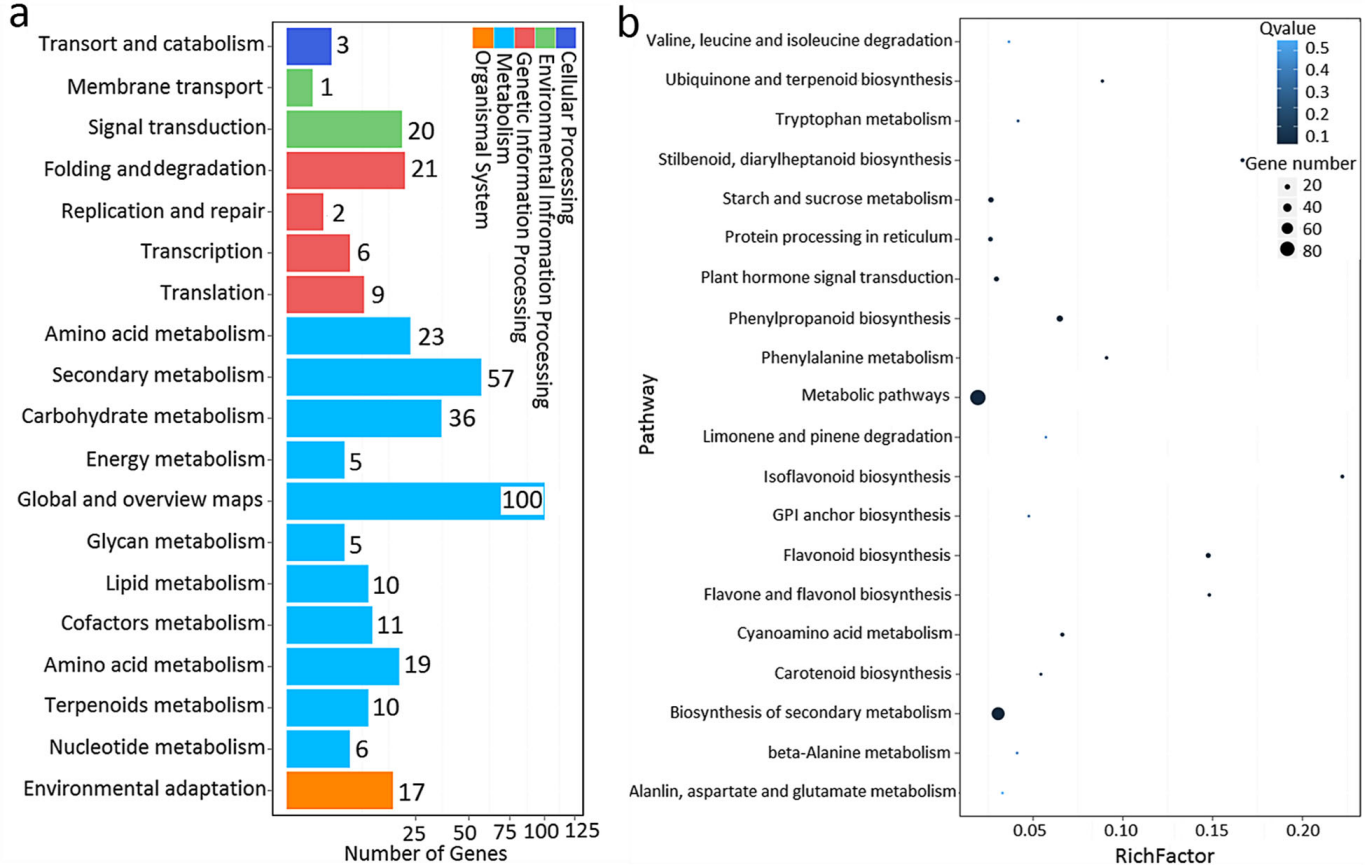
Functional analysis of DEGs in the F/D libraries. **a** KEGG analysis of these DEGs in different metabolic processes. **b** Display of the top 20 enriched pathway terms in F/D libraries. The rich factor was the ratio of differentially expressed mRNAs. Numbers annotated in this pathway term apply to all gene numbers annotated in this pathway term; the greater the rich factor, the greater the degree of enrichment

In terms of the dramatic difference in AR development between the 3-d exposure of libraries D and F, we analyzed their DEGs using the KEGG tool. These DEGs could be classified into five groups, including cellular processing, environmental information processing, genetic information processing, metabolism, and organismal systems. Further analysis showed that 20 DEGs were involved in signal transduction in the group of environmental information processing and 36 DEGs were related to carbohydrate metabolism in the metabolism processing group (Fig. 5a), indicating that they might be the major regulatory pathways during light-dependent AR development. In support, the expression of genes involved in plant hormone signal transduction and the metabolism of starch and sucrose was also altered (Fig. 5b).

**Fig. 6:**
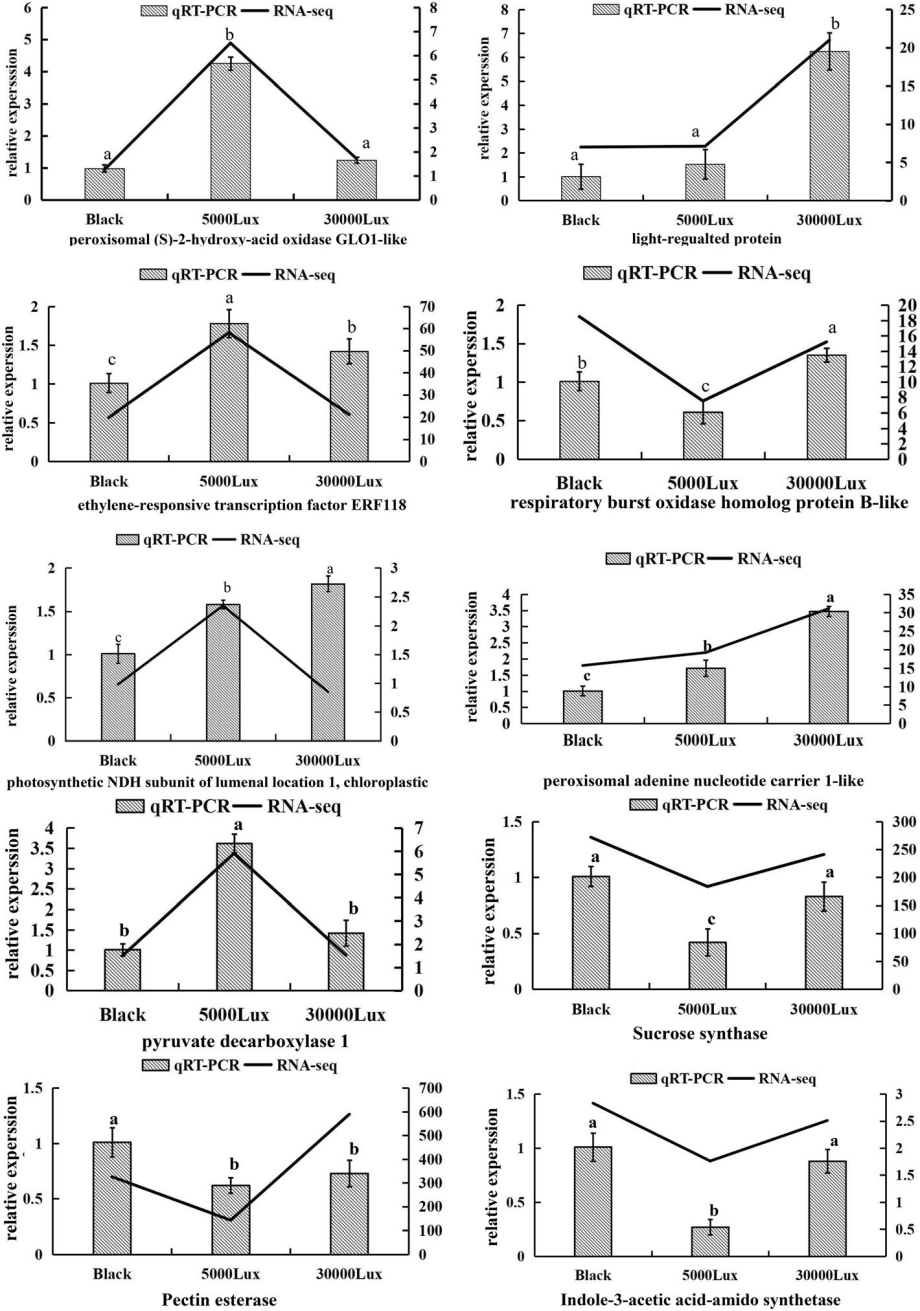
Expression analysis of ten genes under darkness, and under 5000 and 20,000 lx light intensities by qRT-PCR. The genes selected in this study were pectinesterase, peroxisomal adenine nucleotide carrier, indole-3-acetic acid-amido synthetase, ERF118, peroxisomal (S)-2-hydroxy-acid oxidase GLO1, pyruvate decarboxylase 1, respiratory burst oxidase homolog protein B, sucrose synthase light-regulated protein, the photosynthetic NDH subunit

Furthermore, we employed reverse-transcriptase quantitative polymerase chain reaction (qRT-PCR) to confirm the results of RNA-seq. Ten genes, including pectinesterase, peroxisomal adenine nucleotide carrier 1-like, indole-3-acetic acid-amido synthetase, ethylene-responsive transcription factor ERF118, peroxisomal(S)-2-hydroxy-acid oxidase GLO1-like, pyruvate decarboxylase 1, respiratory burst oxidase homolog protein B-like, sucrose synthase, light-regulated protein, photosynthetic NDH subunit of lumenal location 1, which are involved in various processes such as sugar metabolism, IAA signal transduction, energy metabolism, photosynthesis, ethylene signal transduction, and respiratory metabolism, were chosen to investigate their expression under three light intensities (darkness, 5000 lx, and 30,000 lx) by qRT-PCR. Generally, the expression of these genes was similar to that derived from the RNA-seq dataset (Fig. 6).

### Role of sucrose in lotus AR formation

**Fig. 7:**
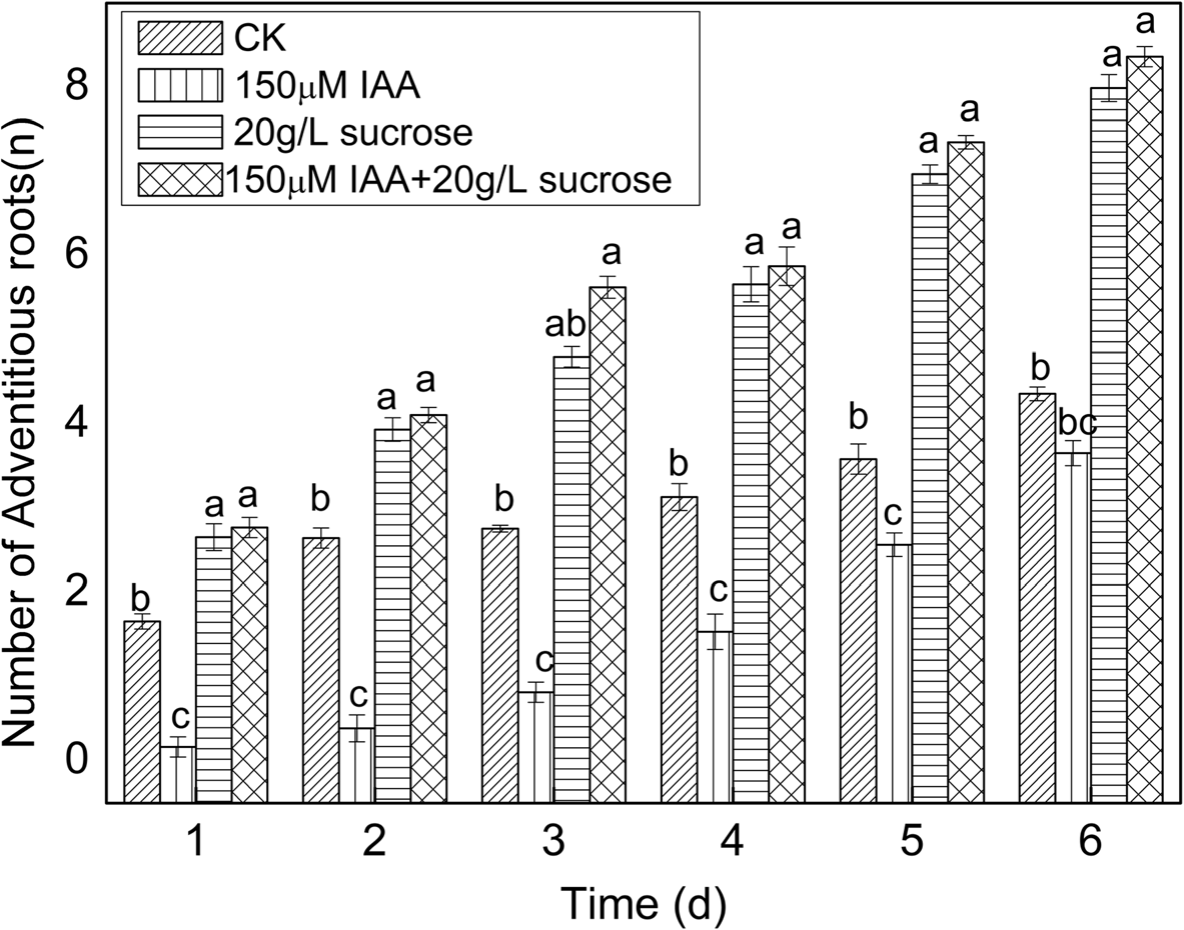
Complementary experiment between IAA and sucrose. Seeds were treated with 150 μM IAA, 20 g/L sucrose, and 150 μM IAA + 20 g/L sucrose for two days with water treatment as the control, and then transferred into water for germination. The number of ARs for each treatment was counted at six time points (days 1, 2, 3, 4, 5, and 6 post-treatment)

**Fig. 8:**
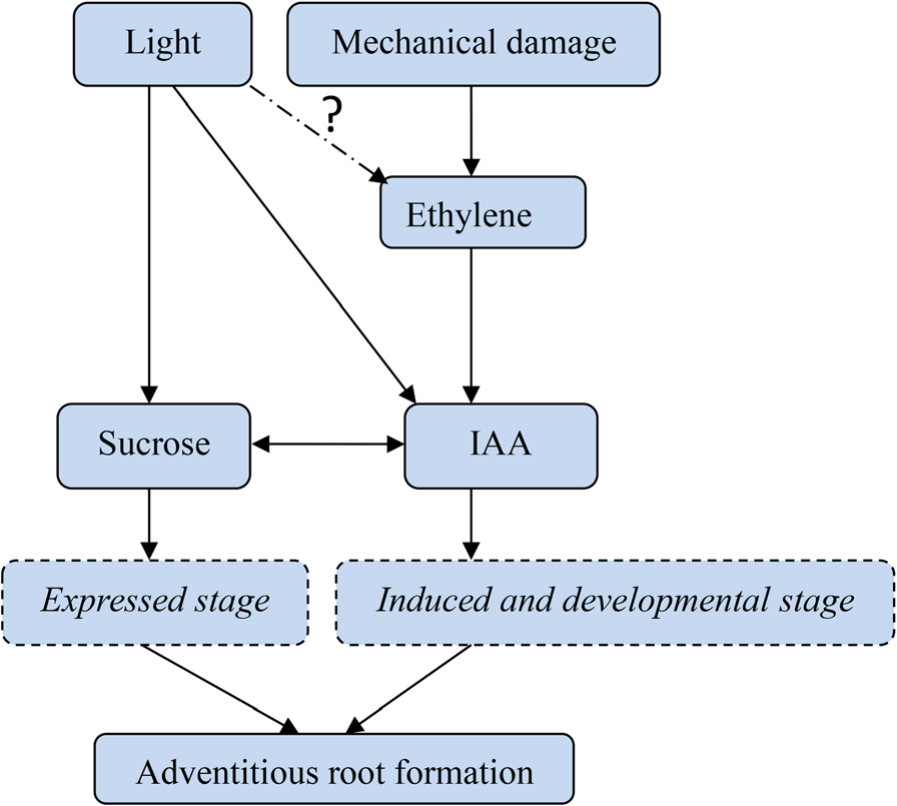
The deduced pathway of sucrose and IAA during lotus AR formation

To analyze the role of sucrose in AR formation, a complementary experiment between IAA and sucrose was carried out under normal light conditions. We found that 60 mg/L sucrose and 150 μmol IAA significantly inhibited AR development, while 20 mg/L sucrose and 10 μmol IAA dramatically promoted the formation of lotus ARs. The inhibition by 60 mg/L sucrose could be compensated by application of exogenous 10 μmol IAA, although no obvious difference was found with control seedlings. Furthermore, exogenous application of 20 mg/L sucrose dramatically increased AR development in seedlings treated with 150 μmol IAA (Fig. 7). According to the change in IAA under various light intensities, IAA was an absolute inducer of ARs in lotus. We observed that ARs could be developed at the induced and developed stage under 150 mg/L IAA treatment (although they could not break out of the epidermis) and remove the inhibitory effect of sucrose. Based on these observations, we believe that sucrose might be involved in the expressed state of lotus AR formation (Fig. 8).

## Discussion

Light (light quality, photoperiod, and light intensity) is an essential environmental factor that affects most plant metabolic processes. There is evidence that light intensity and light quality affect AR development and the photosynthate quantity of *Hypericum perforatum* [40]. Chen et al. (2019) reported that root generation and overall plant development can be dramatically promoted under available light intensity conditions in Haworthia [41]. At the same time, light quality is shown to influence ARs in Coleus [42]. We found that different light intensities have various roles in the formation of ARs in lotus. The seedlings grown under darkness condition could not form ARs, although high light intensity promoted the developmental process of ARs (Fig. 1, Table 1). Interaction of light with other factors is cooperatively involved in root development [43]. Light regulation of AR development is often derived from the sucrose content of photosynthate, and this effect is mainly reflected in the root number [44, 45]. Sorin et al. (2005) found that the role of IAA in regulating AR formation in *Arabidopsis thaliana* is dependent on light conditions. IAA synthesis and accumulation in plants can induce the formation of founder cells of ARs [21]. In our study, we found that IAA content increased with or without light treatment, and that plants under high light intensity had higher IAA content compared with that in plants under low light intensity or darkness (Fig. 2), suggesting that IAA synthesis was affected by light intensity in lotus. Therefore, we believed that ARs formation affected by light was directly regulated by IAA.

Lotus is an important aquatic ornamental plant, and a vegetable in China. ARs are a necessary secondary organ for mainly water and nutrition uptake because no principal roots occur in the plants [32, 46]. Similar to previous reports, three obvious developmental periods, such as induced period of root primordium, developmental period, and expressed period (stages of breaking out epidermis) were found for lotus ARs [32]. We found that many factors including plant hormones [32, 47], mechanical damage [33], and sucrose [data not shown] are all involved in AR development. In addition, several important genes or regulators (miRNAs) have been shown to perform critical roles in AR formation [48]. To monitor the metabolism mechanism regulated by light, gene expression was identified under various light intensities. We found that a large number of genes enhanced expression and decreased expression in the three libraries (D/CK0, E/CK0, and F/CK0), respectively. Based on these datasets, several key genes that demonstrated clear changes in expression were also implored in the F/D libraries (Additional file 1: file S1). Therefore, we concluded that the biological process of AR formation regulated by light was highly complex.

### Role of IAA or sucrose in AR development

Auxin is believed to be a critical hormone that participates in various biological processes such as organogenesis, fruit development, flowering, and adaptation to stresses [39, 49–52]. Auxin metabolism, including auxin synthesis, transport, and homeostasis are known to be involved in the regulation of plant development, such as root formation, shoot development, and reproduction [53]. At the same time, auxin is believed to be a necessary regulator to switch the process from xylogenesis (this process is averse to AR formation) to root development [54]. In the past decades, many important genes relevant to IAA metabolism or the response involved in root formation have been identified [55, 56]. In this study, four libraries were treated with different light intensities were constructed using Solexa technology (Fig. 3), which has proved to be an efficient way to analyze gene expression under certain conditions (March 2011). We found that a total of 4884 genes and 2040 genes demonstrated increased expression and decreased expression, respectively, in the three libraries (D/CK0, E/CK0, and F/CK0, respectively,) (Fig. 4). A total of 28 genes including auxin synthesis, responding (inducing), auxin transporter, and auxin repressed protein was identified in the F/D libraries. However, it was shown that *GH3,* which was responsive for IAA synthesis did not change expression (Table 2), suggesting that another method for IAA synthesis existed according to the change in IAA content. As IAA transporter, auxin is considered an important factor involved in AR development at the induction stages of root primordium development [26, 57, 58]. Interestingly, we observed that the expression of auxin transport-relevant genes such as auxin efflux carrier component 1c and auxin transporter-like protein 2 were not severely affected by light intensity, which indicated that the difference in ARs formation was not a result from IAA transport in the darkness and 20,000 lx light intensity (Table 2).

**Table 2:** Gene expression involved in IAA metabolism in the D/CK, F/CK, and F/D libraries

ID	D/CK0 libraries	F/CK0 libraries	F/D libraries	Function annotation
104,589,706	6.54	–	− 1.83	Auxin induced protein 15A
104,587,078	4.51	–	–	Auxin responsive protein SAUR64
104,602,283	3.98	–	–	Auxin responsive protein SAUR36
104,591,786	3.94	3.82	–	Auxin responsive protein SAUR71
104,600,574	–	3.60	–	Auxin binding protein ABP19a
104,591,143	1.57	–	–	Auxin induced protein X10A
104,599,427	1.51	–	–	Auxin biosynthetic process
104,596,961	1.46	1.52	–	Auxin transporter like protein 2
104,609,680	1.39	–	− 1.07	Auxin transporter like protein 3
104,592,479	1.25	1.24	–	Auxin responsive protein IAA5
104,592,764	−1.05	–	–	Auxin response factor 2
104,610,814	−1.09	–	–	Auxin response factor 6
104,604,767	−1.19	− 1.48	–	Auxin response factor 18
104,601,926	−1.23	− 1.09	–	Auxin efflux carrier component 1c
104,612,448	−1.26	− 1.0	–	Auxin responsive protein IAA4
104,609,555	−1.27	–	–	Auxin induced protein 22B
104,593,954	−1.33	− 1.04	–	Auxin response factor 1
104,592,798	−1.57	− 1.22	–	Auxin response factor 8
104,595,261	− 2.03	−1.58	–	Auxin induced protein AUX22
104,611,678	− 2.24	− 2.70	–	Auxin responsive GH3 gene family
104,586,117	−2.51	− 1.75	–	Auxin responsive protein IAA28
104,595,142	–	− 1.00	–	Auxin responsive protein IAA27
109,113,971	–	− 1.14	− 1.45	Auxin responsive protein SAUR67
109,114,265	–	− 1.16	− 1.50	Auxin responsive protein SAUR68
104,587,994	–	− 1.42	− 1.32	Auxin responsive protein SAUR23
104,608,181	–	− 1.85	− 1.20	Auxin repressed 12.5 kDa protein
109,114,588	–	− 1.94	–	Auxin responsive protein SAUR21
104,595,095	–	− 2.36	–	Auxin induced protein 6B

Plant growth is dependent on cell division, differentiation, and elongation. Sugar (especially sucrose) signals are involved in the above biological processes by inducing many important genes [59–62]. In recent years, sucrose has been found to regulate root growth by increasing root length [63]. Le et al. (2019) reported that application of available concentrations of sucrose in culture medium can significantly promote AR growth [64]. At the same time, sucrose not only affects AR growth, but also influences the substance metabolism of ARs in *Echinacea pallida* [65]. Takahashi et al. (2003) showed that the role of sucrose on AR formation is similar to that of hormones and that the induction stage of AR development is strictly controlled by sucrose [66]. In this study, we found that several important genes related to sucrose synthesis had enhanced expression in the F/D libraries, and the content of sucrose was higher than that in darkness. Sucrose synthase and sucrose-phosphatase were the critical enzymes in sucrose synthesis (Fig. 5, Table 3). It has been shown that the expression of these genes can lead to an increase in the sucrose content [67]. The change in sucrose content by modifying the activity of sucrose synthase and sucrose-phosphatase is beneficial for maintaining fruit storage quality [68, 69]. We observed that the expression of genes encoding sucrose synthase and sucrose-phosphatase was enhanced by 3.91- and 1.29-fold in the F/D libraries (Table 3). A high correlation was found between gene expression and sucrose content, suggesting that sucrose metabolism was necessary for AR development in lotus. In addition, we observed that a complementary phenomenon existed between IAA and sucrose during AR formation, which could explain how IAA and sucrose cooperatively regulated lotus AR formation according to the change in IAA and sucrose content. A crosstalk exists is known to exist between ethylene and auxin in controlling root development. Exogenous application of auxin can increase ethylene content, and plants treated with ethephon also improve the content of endogenous IAA content [22, 24, 70]. Therefore, we believe that a new model of interaction between sucrose and IAA is involved in lotus root development.

**Table 3:** Gene expression involved in sucrose, glucose, and fructose metabolism in D/CK, F/CK, and F/D libraries

ID	D/CK0 libraries	E/CK0 libraries	F//CK0 libraries	Function annotation
Sucrose metabolism				
104606206	4.07	3.43	3.91	Sucrose synthase 2
104590896	3.38	3.37	–	Sucrose-phosphate synthase 2
104607575	1.64	2.34	2.18	Sucrose transport protein SUC4
104591964	1.61	1.94	1.29	Galactinol--sucrose galactosyltransferase
104595245	−1.47	–	–	VIN3-like protein 1
104599026	−1.01	–	–	Sucrose nonfermenting 4
104605199	−1.03	–	1.29	Sucrose-phosphatase 2
104590476	−1.93	− 1.47	−1.37	Sucrose synthase
104586132	−2.15	− 2.98	−1.99	Galactinol--sucrose galactosyltransferase 6
104612986	−2.50	−2.91	− 2.80	Galactinol--sucrose galactosyltransferase 2
104607856	−3.17	−2.82	− 3.14	Sucrose synthas
Glucose metabolism				
104613182	3.84	3.37	2.73	Facilitated glucose transporter
104611373	3.78	3.23	4.54	Sugar transport protein 14
104596455	2.64	–	–	Glucose-1-phosphate adenylyltransferase
104586894	2.09	1.65	1.66	Sugar transporter ERD6-like 5
104589792	1.83	1.83	1.57	Glucosyltransferase
104590530	1.52	–	–	UDP-glycosyltransferase 84B2-like
104606042	1.39	1.16	1.34	Phosphoglycerate kinase
104598555	1.15	1.71	1.20	L-lactate dehydrogenase B
104599589	1.09	1.08	1.24	6-phosphogluconolactonase 2
104597981	−1.00	–	–	UDP-glucose 6-dehydrogenase 1
104605632	−1.09	–	–	Glucose-induced degradation protein 8
104601180	−1.14	− 1.10	−1.12	Cytosolic enolase 3
104605346	−1.24	–	–	Hexokinase-2
104601854	−1.32	–	–	Sugar transporter ERD6
104590451	−1.52	− 1.26	−1.36	UTP-glucose-1-phosphate uridylyltransferase
104591632	−1.63	−1.45	–	Glucose-6-phosphate 1-dehydrogenase
104601106	−1.72	− 1.62	−1.19	UDP-glucose 6-dehydrogenase 4
104611174	−1.80	–	–	Pyruvate kinase
104593418	−2.86	− 2.54	−2.83	Glucose-6-phosphate 1-epimerase
104598792	−2.96	–	–	UDP-glucose 6-dehydrogenase 5
104592969	−3.63	− 3.04	−3.67	Glucose-6-phosphate 1-dehydrogenase
Fructose metabolism				
104602041	2.27	1.71	1.87	Fructose-bisphosphate aldolase 1
104595011	−1.29	–	–	6-phosphofructo-2-kinase
104606877	−1.77	− 1.58	−1.76	Pyrophosphate-fructose 6-phosphate 1-phosphotransferase

## Conclusion

In this study, we observed that lotus ARs were regulated by light intensity. Therefore, we analyzed genome-wide gene expression by RNA-seq and determined that 1739, 1683, and 1462 genes were upregulated and 1533, 995, and 834 genes were downregulated in the D/CK0, E/CK0, and F/CK0 libraries, respectively. We observed that 20 DEGs were involved in signal transduction, and 36 DEGs were related to carbohydrate metabolism. In the F/D libraries, 28 genes relevant to auxin synthesis, transport, response, or binding were found to participate in AR development, and 24 genes related to sucrose metabolism, glucose metabolism, and fructose metabolism changed their expression. According to changes in IAA and sucrose content, we believe that IAA and sucrose cooperatively regulate lotus AR formation.

## Methods

### Material preparation

In this study, Taikong 36, which was bred by the research base of the Guangchang space lotus (this species of lotus, *Nelumbo nucifera* Gaertn., has been deposited in a publicly available herbarium), was selected for all experimental analyses as there were abundant material resources for this plant. Taikong 36 was harvested from Guangxi province, and grown in the experimental field of the aquatic vegetables research group of Yangzhou University (latitude: 119.42, longitude: 32.39, altitude: 12 m), Southeast China with conventional management in spring. In the growing season, the temperature was 30 ± 5 °C/day and 25 ± 5 °C/night, and the average water depth of field (the plant must be maintained in the water) was 30 ± 10 cm. The seeds were harvested after 30 d of flowering and kept in the warehouse under normal temperature conditions. The high activity of lotus seeds can be maintained for 10 years.

### Paraffin section experiments

The seed coat of Taikong 36 lotus was broken and soaked in water for germination. The seedlings of lotus were exposed to various light intensities including darkness, and 5000, and 20,000 lx at a temperature of 30 °C/day and 20 °C/night. Lotus hypocotyls were chosen at day 0, 1, 2, 3, 4, and 5 post-treatment. Hypocotyls sections of 2 mm × 2 mm × 2 mm (length × width × height) were prepared and placed into a small container with approximately 10 mL free fatty acid fixing fluid (at a 20:1 volume of free fatty acid fixing fluid to each sample). A vacuum condition in a the small container was made with a syringe, and the samples of these treatments were transferred to this vacuum environment for 10 s, and then gas was exchanged for 10 min. The above process was repeated three times at 25 °C overnight. Then, various concentrations of ethanol (50, 60, 70, 80, 90, and 100%) were prepared and used to treat samples for 20 min sequentially. These dehydrated samples were put into a mixed solution (pure xylene: absolute ethanol; 1:1), and then transferred to pure xylene for approximately 20–30 min. The samples wrapped in paraffin were placed in a container for at least 12 h at normal temperature, and then transferred into thawed paraffin wax for 18–24 h for preparing paraffin blocks. An incisive slicer was used to prepare a 10 μm thick wax tape, which was then placed on the glass slide. The samples on the glass slide were treated in turn with xylene, mixed solution (pure xylene: absolute ethanol; 1:1), and absolute ethanol for approximately 10 min. Finally, these slides were dried in air under normal temperature conditions, and the tissues on the slide were identified using an optical microscope.

### RNA-sequencing analysis

For this experiment, the lotus seed coat was broken for available uptake of water, germinated at 28–30 °C, and then transferred into a container with 5 cm of water depth for growth. The one-leaf seedlings were placed into three light intensities, including darkness, 5000 lx, and 20,000 lx for AR analysis. The materials of these treatments were chosen at day 0 (CK0), and day 3 (library D was constructed from samples under darkness; library E was constructed from samples from 5000 lx treatment; and library F was constructed from samples from 20,000 lx treatment). The total RNA of hypocotyls was prepared and digested with DNase I for purification. Approximately 3 μg of RNA from each sample was used for library construction. Sample preparation kits for Illumina gene expression were used for CK0, D, E, and F library construction. Processing of libraries were completed as previously described [32]. The detailed work was completed by the Beijing Institute of Genomics (BIG) with a special construct.

### Screening of differentially expressed genes

DEGs of differential libraries were screened as previously described method [71, 72]. Further analysis of reads derived from all libraries was carried out by the NOISeq technique [32]. The log2 (fold-change) M was used as the relative expression of genes in each library, and the noise distribution model was built according to the absolute value of difference (N). To identify whether gene A was differentially expressed, the average expression in control group (control_avg) and treatment group (treat_avg) was first computed, and then the values of different expressing change and “N” were obtained according to the data of fold change (MA = log2 ((treat_avg)/(control_avg))) and the absolute value of difference (NA = |control_avg - treat_avg|). Finally, gene A was considered as a DEG if MA and NA measured by probability value diverged significantly from the noise distribution model. Totally, if the fold change of expressed gene A was greater than two and the divergence probability was greater than 0.8 then gene A was accepted as a DEG.

### Functional analysis of the DEGs

The DEGs in each library involved in molecular function, cellular component, and biological process were annotated using the Gene Ontology (GO) tool. The biological function of DEGs was obtained by comparing results with the National Center for Biotechnology Information (NCBI) database. The number of DEGs involved in the above three ontologies was computed after comparison with the GO database (http://www.geneontology.org/), and then the DEGs were collected together as GO terms by hypergeometric test. In addition, the KEGG tool was used to identify which metabolic processes these DEGs were enriched in.

### Relative expression analysis

The relative expression of several important genes was documented to identify the changes in metabolism under various light intensity treatments. The lotus seed treatment and cultivation conditions were the same as mentioned above. The samples were collected at 0 (germinated seeds) and day 3 (germinated seeds that were cultivated for 3 days in darkness, and under 5000 and 20,000 lx conditions). qRT-PCR was applied to monitor changes in gene expression, as previously described [11; 69]. The RNA extraction mini kit (QIAGEN, Germany) was used to obtain the total RNA of lotus hypocotyls, and DNA contamination was removed by DNaseI. First Strand cDNA Synthesis Kit (Fermentas, USA) [73] was used to synthesize cDNA (approximately 3–5 μg of total RNA). The transcriptional level was investigated using SYBR Green Master Mix (Tiangen, China) on an Mx 3000P machine (STRATAGENE, http:// www.stratagene.com) [61] with three repeated experiments. The primers of selected genes were derived from the NCBI database, and β-Actin was used as the reference gene to measure gene expression. The primers for β-Actin were: upstream, 5′-AACCTCCTCCTCATCGTACT-3′, and downstream, 5′-GACAGCATCAGCCATGTTCA-3′; the primers of the chosen genes are listed in Additional file 1: Table S2. The total volume of the PCR reaction system was 25 μL including 12.5 μL Premix Ex Taq II (TliRNaseH Plus) (2×) SYBR (Tiangen, China), 10 μM each of forward and reverse primers, 2 μL cDNA solution, and 8.5 μL distilled water. The PCR program was run at 94 °C for 30 s, followed by 40 cycles of 95 °C for 5 s and 55 °C–60 °C for 60 s. The data analysis was performed using the 2^-△△Ct^ method [48].

### Analysis of IAA content

The seeds of lotus were broken and then put into a container with 5 cm water in depth for germination at 26 °C for approximately 4–5 d. The germinated seeds were transferred into four light intensities for continuous growth (under darkness, and under 5000, 15,000, and 30,000 lx); hypocotyls of lotus seedlings were selected at 0, 2, 4, 6, 8, and 10 d of each treatment for IAA identification according to the following procedure. First, the hypocotyls of each treatment were put into liquid nitrogen to grind into powder, and then 50 mg of sample powder was transferred into a 2 mL centrifuge tube, which was pre-cooled in advance. Next, approximately 500 μL of extraction reagent (V/V/V: isopropyl alcohol: water: concentrated hydrochloric acid = 2:1: 0.002) was added to the tuber. The tubers were oscillated at 4 °C for 30 min at 100 rpm, and 1 mL dichloromethane was added. After shaking at 4 °C for 30 min, the mixed solution was centrifuged at 12,000–13,000 rpm at 4 °C for 5 min. The lower layer of the supernatant (900 mL) was extracted and dried with nitrogen; 100 mL of filtered methanol was added to dissolve the dry powder. The sample (50 mL) was injected into the C18 column of liquid chromatography (Sigma, Germany) for IAA identification.

### Complementary effect of sucrose on IAA treatment of lotus ARs

Lotus seed was broken for available uptake water, and then was divided into four groups. The seeds of the first group were placed in water for germination, and the second group was treated with 150 μM IAA for 2 days and then transferred to water for germination. The seeds of the third group were put into 20 g/L sucrose for 2 days and then transferred into water, and the seeds of the fourth group were placed into the solution with 20 g/L and 150 μM IAA for 2 days. The conditions of germination, including temperature, water depth, and light intensity, were the same as described above. After germination of seeds, the number of ARs was counted with an interval of 1 day within 6 days. The experiment was repeated three times.

## Supplementary information

**Additional file 1: Fig S1.** Analysis of sequencing data saturation in CK, D, and E and F libraries. a. C0 library. b. D library. c. E library. d. F library. Differentially expressed genes in D/CK, E/CK, F/CK, and F/D. libraries. **Table S1.** Information on tags obtained by RNA-seq technology in all the libraries. **Table S2.**The primers of genes used for mRNA level analysis qRT-PCR method.

## Data Availability

The materials of all the experiments were supported by the aquatic vegetable Laboratory of Yangzhou University. Detailed data has been deposited in the NCBI database (**Biosample number**, CK0_1 ~ CK0_3: SAMN13000907 ~ SAMN13000909, CK0_1–CK0_3: SAMN13000910 ~ SAMN13000912; E_1 ~ E_3: SAMN13000913 ~ SAMN13000915; E_1 ~ E_3: SAMN13000916 ~ SAMN13000918; **Bioproject number**: 576670).

## Abbreviations

ARs: Adventitious roots
IAA: Indole-3-acetic acid
DEGs: Differentially expressed genes
PCR: Polymerase chain reaction
qRT-PCR: Reverse-transcriptase quantitative PCR
