# Supplementary material for: Gene expression profiling reveals the effects of light on adventitious root formation in lotus seedlings (Nelumbo nucifera Gaertn.)
Source: BMC Genomics. 2020 Oct 12;21:707. doi: 10.1186/s12864-020-07098-5 (PMC7552355; doi:10.1186/s12864-020-07098-5)
Supplement: Supplementary file 1 — Additional file 1: Fig S1. Analysis of sequencing data saturation in CK, D, and E and F libraries. a. C0 library. b. D library. c. E library. d. F library. Differentially expressed genes in D/CK, E/CK, F/CK, and F/D. libraries. Table S1. Information on tags obtained by RNA-seq technology in all the libraries. Table S2.The primers of genes used for mRNA level analysis qRT-PCR method. [file 12864_2020_7098_MOESM1_ESM.zip › Supplementary table.S1.docx]

| Sample | Raw Data Size (bp) | Clean Data Size (bp) | Clean Data Rate (%) | Total Reads | Total Mapped Reads (%) | Unique Match(%) |
| --- | --- | --- | --- | --- | --- | --- |
| CK_1 | 1206833900 | 1203977300 | 99.76 | 24079546 | 84.13 | 74.14 |
| CK_2 | 1206848000 | 1204044250 | 99.76 | 24080885 | 83.85 | 73.94 |
| CK_3 | 1206854850 | 1204094600 | 99.77 | 24081892 | 87.14 | 76.6 |
| D_1 | 1206852050 | 1178053350 | 97.61 | 23561067 | 87.68 | 76.87 |
| D_2 | 1197310950 | 1195880100 | 99.88 | 23917602 | 87.59 | 76.27 |
| D_3 | 1206828700 | 1204435050 | 99.8 | 24088701 | 88.08 | 76.81 |
| E_1 | 1191344350 | 1162710150 | 97.59 | 23254203 | 85.29 | 75.25 |
| E_2 | 1298924250 | 1103638900 | 84.96 | 22072778 | 84.98 | 74.9 |
| E_3 | 1206855550 | 1204116600 | 99.77 | 24082332 | 86.2 | 75.97 |
| F_1 | 1193337500 | 1165783500 | 97.69 | 23315670 | 87.19 | 76.45 |
| F_2 | 1188996950 | 1187638750 | 99.88 | 23752775 | 86.62 | 76.02 |
| F_3 | 1206849200 | 1204335650 | 99.79 | 24086713 | 87.8 | 76.98 |
