# Supplementary figures and images for: Gene expression profiling reveals the effects of light on adventitious root formation in lotus seedlings (Nelumbo nucifera Gaertn.)
Source: BMC Genomics. 2020 Oct 12;21:707. doi: 10.1186/s12864-020-07098-5 (PMC7552355; doi:10.1186/s12864-020-07098-5)

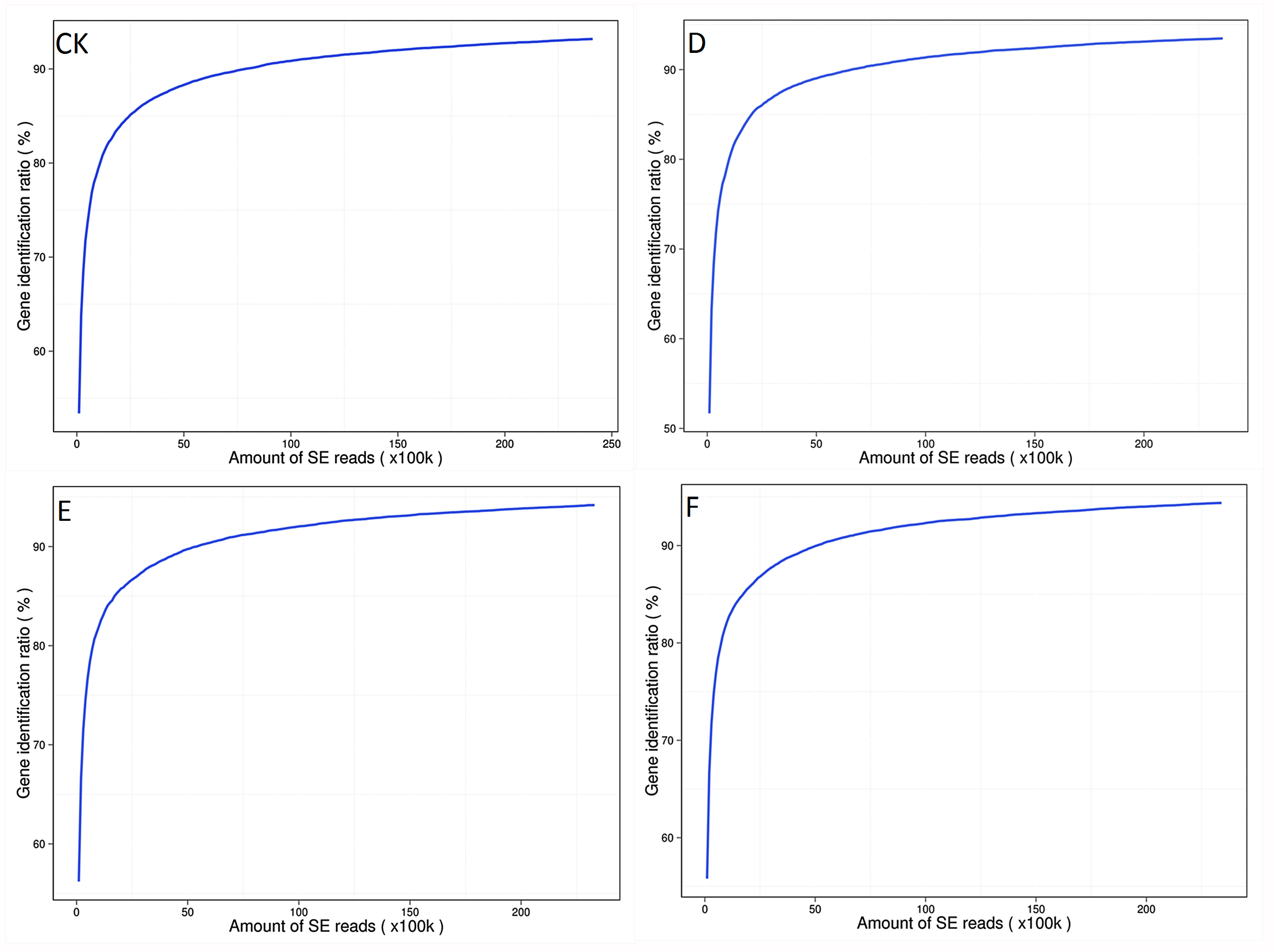

Supplement: Supplementary file 1 — Additional file 1: Fig S1. Analysis of sequencing data saturation in CK, D, and E and F libraries. a. C0 library. b. D library. c. E library. d. F library. Differentially expressed genes in D/CK, E/CK, F/CK, and F/D. libraries. Table S1. Information on tags obtained by RNA-seq technology in all the libraries. Table S2.The primers of genes used for mRNA level analysis qRT-PCR method. [file 12864_2020_7098_MOESM1_ESM.zip › Supplementary fig.S1.tif]
