# Supplementary material for: Gene expression profiling reveals the effects of light on adventitious root formation in lotus seedlings (Nelumbo nucifera Gaertn.)
Source: BMC Genomics. 2020 Oct 12;21:707. doi: 10.1186/s12864-020-07098-5 (PMC7552355; doi:10.1186/s12864-020-07098-5)
Supplement: Supplementary file 1 — Additional file 1: Fig S1. Analysis of sequencing data saturation in CK, D, and E and F libraries. a. C0 library. b. D library. c. E library. d. F library. Differentially expressed genes in D/CK, E/CK, F/CK, and F/D. libraries. Table S1. Information on tags obtained by RNA-seq technology in all the libraries. Table S2.The primers of genes used for mRNA level analysis qRT-PCR method. [file 12864_2020_7098_MOESM1_ESM.zip › Supplementary table.S2..doc]

| Primers | Forward primers (5′-3′) Reverse primers (5′-3′) |
| --- | --- |
| Pectinesterase | ACCGCCAGTTCTACCGAGACAC GCCGCTACGATCTTACAGTTGTGG |
| Peroxisomal adenine nucleotide carrier | CCGTTGGATCACCTTCGAGATTGG ATCGGAGACACGGCCACTGAG |
| Indole-3-acetic acid-amido synthetase | TTGCTACCGCTAATCGCCTAATCC TGTCAATTCCAGCCACCAAGGAC |
| ERF118 | CAGCACTCATCGACGACCAACG AGGTCCATCAACGCATCGAATGTC |
| Peroxisomal (S)-2-hydroxy-acid oxidase GLO1 | TGCTGAAGAGGCCGCTGAGG AGACGATGGTGTATTGTGGTTGCC |
| Pyruvate decarboxylase 1 | GATGGTGAAGCTGGTGTGAGGAAG GAGTGAGCGGCAACCACATAGTG |
| Respiratory burst oxidase homolog protein B | GCTGTTCGTGTGCTTGATTGTCG GCTGACACTGGCTTCTCATCTGG |
| Sucrose synthase | GTTGCAGGTAGACGGTTCACTTCC GGTTCGCCACGCACACTTAGAG |
| Light-regulated protein | GCGACGATGAGCACCGATTGAG GCGAGATCGAAGCCATTGGAGAAG |
| Photosynthetic NDH subunit | TGCCACCTACCAGGACCACTG GCCGACAGCATACTTACGAGGTC |
| β-actin | GACTCTGGTGATGGTGT CACTTCATGATGGAGTTGT |
